# The Role of Parental Overreactivity and Laxness in Diabetes Outcomes Among Children and Adolescents With Type 1 Diabetes

**DOI:** 10.1155/jdr/2480622

**Published:** 2026-02-27

**Authors:** Alessa Thomas, Su-Jong Kim-Dorner, Olga Kordonouri, Bettina Heidtmann, Thomas M. Kapellen, Simone von Sengbusch, Roland Schweizer, Karin Lange, Heike Saßmann

**Affiliations:** ^1^ Department of Medical Psychology, Hannover Medical School, Hannover, Germany, mh-hannover.de; ^2^ Diabetes Centre for Children and Adolescents, Children′s Hospital AUF DER BULT, Hannover, Germany; ^3^ Pediatric Endocrinology and Diabetes, Wilhelmstift Catholic Children′s Hospital, Hamburg, Germany; ^4^ Department of Pediatrics, University of Leipzig, Leipzig, Germany, uni-leipzig.de; ^5^ MEDIAN Children′s Hospital “Am Nicolausholz”, Naumburg, Germany; ^6^ Clinic for Pediatric and Adolescent Medicine, University Hospital Schleswig-Holstein, Lübeck, Germany, uk-sh.de; ^7^ Pediatric Endocrinology and Diabetology, University Children′s Hospital Tübingen, Tübingen, Germany

**Keywords:** children and adolescents, diabetes distress, laxness, overreactivity, parenting behavior, self-management behavior, Type 1 diabetes

## Abstract

**Introduction:**

Parental disciplinary behaviors, such as *overreactivity* and *laxness*, can negatively affect children′s behavior and well‐being. We examined ove*rreactivity* and *laxness* in relation to parental emotional well‐being and the behavioral, glycemic, and psychological outcomes of children and adolescents with T1D.

**Methods:**

A cross‐sectional survey was conducted at five pediatric diabetes centers. Parents provided demographic and clinical information and completed questionnaires assessing parenting behavior, diabetes‐specific distress, and depressive symptoms. Children reported their frequency of glucose checking and diabetes distress. Correlation and regression analyses were used. Child age was stratified into three groups (7–10, 11–14, and 15–18 years) for subgroup analyses.

**Results:**

A total of 677 parents (mean age = 43.9 [±6.3] years, 77.1% mothers) and 654 children (mean age = 12.4 [±2.8] years, 44.7*%*female, diabetes duration = 5.4 [±3.8] years) participated. With increasing child age, parents reported less *overreactivity* (*r* = −0.114, *p* = 0.003) and more *laxness* (*r* = 0.104, *p* = 0.007). Higher *overreactivity* and *laxness* were associated with greater parental diabetes distress (*r* = 0.335, *p* < 0.001; *r* = 0.119, *p* < 0.01) and more depressive symptoms (*r* = 0.314, *p* < 0.001; *r* = 0.100, *p* < 0.01). After adjusting for demographic and clinical variables, both disciplinary behaviors were significant factors in child diabetes distress, and *laxness* predicted less frequent glucose checking. Age‐stratified analyses showed that *overreactivity* was linked to greater diabetes distress in children aged 7–10 years. *Laxness* was associated with fewer glucose checks in the 7–10 and 15–18‐year groups and with higher HbA1c and diabetes distress in adolescents aged 15–18 years.

**Conclusions:**

Parents appear to use more *overreactivity* with younger children and more *laxness* as children age. Both behaviors are associated with poorer diabetes‐related outcomes. Supporting effective parenting practices may improve psychological well‐being and self‐management of youth with T1D.

## 1. Introduction

Type 1 diabetes (T1D) is among the most common chronic conditions in children, with incidence rates rising worldwide [1]. Managing T1D requires continuous and complex self‐care, including blood glucose monitoring, insulin administration, dietary regulation, and physical activity adjustments to maintain optimal glucose levels and avoid severe acute and long‐term complications [2, 3]. Effective diabetes management in children and adolescents depends not only on medical care but also on consistent and supportive parental involvement, especially as self‐care responsibilities gradually shift from parents to older children and adolescents during this developmental period [4].

Parenting a child with a chronic condition presents unique challenges. Studies have found that parents of children with T1D experience greater parental distress than those of healthy children [5]. The additional daily tasks associated with T1D care [6, 7] coupled with constant anxiety and worries about acute and long‐term complications can disrupt typical parenting patterns. This may further influence how parents respond to their child′s diabetes‐related behaviors, especially during moments of conflict, nonadherence, or emotional distress. Previous studies show that an authoritative parenting style, generally characterized by warmth, consistency, effective communication, and appropriate monitoring, is associated with better diabetes outcomes, including improved glycemic control and adherence [8]. In contrast, harsh, intrusive, permissive, or inconsistent parenting has been linked to poorer glycemic control, reduced adherence, greater diabetes distress, and reduced mental well‐being in children with T1D [8–11]. These ineffective parenting behaviors can heighten stress for both parents and children and increase family conflict and dysfunctional interactions [5, 7, 12, 13].

Despite this, there is a limited understanding of how specific dysfunctional parenting behaviors, rather than broad parenting styles, affect diabetes outcomes. Parenting practices refer to the specific behaviors that parents use to guide, socialize, and support their children [14]. Among these, parental disciplinary strategies play an important role in shaping children′s behavioral, emotional, and psychosocial development [15, 16]. Two maladaptive practices, *overreactivity* and *laxness*, are considered to be core dimensions of dysfunctional parenting because of their negative effects on children′s behavior and well‐being. *Overreactivity* involves harsh, coercive, and emotionally charged parental responses in conflict situations and has been linked to behavioral problems in children and adolescents [17, 18]. *Laxness*, by contrast, refers to inconsistent or overly lenient parenting characterized by difficulty setting limits and enforcing rules in disciplinary situations. These behaviors in parents may be particularly consequential in the context of diabetes management, with potential implications for both metabolic control and psychological well‐being in youth with T1D. However, few studies have examined these specific parental behaviors in families of children with T1D.

As parenting practices and diabetes management responsibilities evolve with child age, the impact of these behaviors may differ across developmental stages. Research shows that parents of adolescents often adapt more lax parenting practices to support age‐appropriate autonomy [19]. However, such shifts may be problematic for adolescents with T1D, considering consistent monitoring and guidance is essential [20]. To date, no studies have examined the distinct effects of *overreactivity* and *laxness* on biomedical, behavioral, and psychological outcomes among children and adolescents with T1D.

The present study addresses this gap by examining the two most frequently used parental disciplinary practices, *overreactivity* and *laxness*, among parents of youth with T1D. We assess whether these behaviors are associated with diabetes management adherence, clinical outcomes, and psychological well‐being in school‐aged children and adolescents with T1D. We hypothesize that higher levels of *overreactivity* and *laxness* will be linked to less frequent glucose monitoring, poorer glycemic control (higher HbA1c levels), and greater diabetes‐related distress. We also explore whether these associations vary across age groups. By focusing on specific and measurable parenting behaviors, this study is aimed at clarifying how parents′ responses during everyday conflict situations may influence both medical and psychological outcomes in children and adolescents with T1D.

## 2. Materials and Methods

### 2.1. Participants

Participants were parents and their school‐aged children (7–18 years) with at least 1 year of T1D duration, who were visiting one of the five pediatric diabetes centers in Germany (Hannover, Hamburg, Lübeck, Bad Kösen, and Tübingen). The accompanying parent had to be currently living with the child. If both parents were present, only one parent per family completed the questionnaire. Children were instructed to complete the questionnaire independently. Both parent and child had to be able to read and write in German in order to provide verbal consent and complete the questionnaires.

### 2.2. Study Design and Procedures

This multicenter cross‐sectional survey was conducted between January 2021 and December 2022. During routine check‐ups, trained nurses at the participating centers approached all eligible families, providing information about the study both orally and in writing. After giving verbal consent, parents and children independently completed anonymous, self‐administered paper questionnaires and deposited in a collection box. This study was approved and monitored by the Ethical Committee on Human Studies at Hannover (No. 9359_BO_K_2020, September 30, 2020) in accordance with the Declaration of Helsinki.

### 2.3. Measures

#### 2.3.1. Demographics and Diabetes‐Specific Data

Parents provided demographic information, including their gender, age, country of birth, living situation, employment status, and education. Clinical information about the child′s diabetes included age at diagnosis, diabetes duration, current use of an insulin pump and continuous glucose monitoring (CGM), time in range (TiR) (70–180 mg/dL based on the last 14 days), and HbA1c at the current visit. HbA1c and TiR values were recorded by the participant based on information provided by the attending nurse or physician. Children reported the average number of glucose checks they performed daily. No medical records were accessed, and no additional examinations were conducted as part of this study.

#### 2.3.2. Psychological Measures

*Parenting practices*, overreactivity and laxness, were assessed using the 13‐item German short version of the Parenting Scale (PS) (*Erziehungsfragebogen-Kurzform* [*EFB-K*]) [21], adapted from the original “PS” by Arnold et al. [22]. Each item describes a hypothetical disciplinary situation and is rated on a 7‐point Likert scale with higher scores indicating more negative parenting behaviors in the last 2 months. High overreactivity scores reflect harsh, irritable, or angry responses. Similarly, high laxness scores indicate permissive and inconsistent discipline and ineffective limit‐setting. The *EFB-K* yields separate mean scores for overreactivity and laxness, which were used in this study. Suggested cutoffs for dysfunctional parenting practice are ≥ 4.34 for overreactivity and ≥ 3.43 for laxness. The scales have been widely used and several studies supported these two factors [23, 24] and validity in parents of both younger children and adolescents [23, 25, 26]. In the current study sample, internal consistency for *overreactivity* and *laxness* scales was *α* = 0.76 and 0.73, respectively.

*Diabetes-related emotional burden* was assessed using the *Problem Areas in Diabetes* (*PAID*) [27, 28] which measures self‐reported level of diabetes‐specific distress in children with T1D and their parents. The German versions used were PAID‐C for children (7–12 years), PAID‐T for adolescents (13–18 years), P‐PAID‐C for parents of children, and P‐PAID‐T for parents of adolescents [29, 30]. Respondents rated the degree to which each item had been a problem over the past month on a 6‐point Likert scale (1 =  ^“^ *not* *a* *p* *r* *o* *b* *l* *e* *m* ^”^ to 6 =  ^“^ *s* *e* *r* *i* *o* *u* *s* *p* *r* *o* *b* *l* *e* *m* ^”^) with higher scores indicating greater distress. The mean scores were used to represent average diabetes distress. Internal consistencies in our sample were great: *α* = 0.88 for PAID‐C, *α* = 0.91 for PAID‐T, and *α* = 92 for both P‐PAID‐C and P‐PAID‐T.

*Health-related quality of life* (*HRQoL*) in children was assessed with the *KIDSCREEN-10 index* [31–33]. It provides a global index of *HRQoL* covering physical, psychological, and social aspects in healthy and chronically ill children aged 8–18 years. Ten items are rated on a 5‐point Likert scale, with higher scores indicating higher *HRQoL* over the past week. Rasch *T*‐scores were generated and used in analyses. In our sample, the KIDSCREEN‐10 index demonstrated good internal consistency, with a Cronbach′s *α* of 0.82.

*Depressive symptoms* in parents were measured using the *Patient Health Questionnaire-9* (*PHQ-9*). The *PHQ-9* measures depressive symptoms using nine items corresponding to the DSM‐IV criteria for major depressive disorder [34]. Each item is rated on a 4‐point Likert scale (0–3), yielding a total score from 0 to 27. Higher scores indicate a higher probability of depression, with cutoffs of 0–4 for *no symptoms*, 5–9 for *mild*, and ≥ 10 for *moderate to severe depressive symptoms* [34]. Internal consistency in this sample was *α* = 0.85.

### 2.4. Data Analysis Plan

All analyses were conducted using IBM SPSS Statistics for Windows, Version 29.0.1.0 (IBM Corp, Armonk, New York, United States). Figures were created using excel (Microsoft Office Professional Plus 2019).

Descriptive statistics are presented as mean (M) ±  standard deviation (SD)standard deviation (SD). Group differences were analyzed using independent *t*‐tests for two groups and one‐way ANOVA for more than two groups. Statistical significance was set at *p* < 0.05 (two‐tailed). Pearson′s correlations were used to examine relationships between continuous variables, with coefficients categorized as small (0.1–0.29), moderate (0.3–0.49), and large (≥ 0.5) for interpretation [35].

Three separate hierarchical regression analyses were conducted to examine the influence of parenting behaviors on children′s behavioral, glycemic, and emotional outcomes. Dependent variables were glucose checks, HbA1c, and diabetes‐related emotional distress. Independent variables (IVs) were selected based on prior literature and significant correlations with dependent variables in our data (*p* < 0.05). For the glucose check model, IVs included child age and gender, diabetes duration, insulin pump and CGM use, parental education, and child′s *HRQoL*. For the HbA1c and diabetes distress models, parental marital status was additionally included. *Overreactivity* and *laxness* were then entered in all three models. Finally, to examine developmental differences, separate backward regression analyses were conducted for primary school children (7–10 years), early adolescents (11–14 years), and older adolescents (15–18 years).

A single missing item on the *EFB-K*, *PAID*, or *PHQ-9* was imputed using the respondent‐specific mean of the remaining answered items. If more than one item was missing on a given scale, no total or mean scores were computed. Parents with more than one missing item on the *EFB-K* were excluded from the analyses.

## 3. Results

Of the 921 families approached, 802 agreed to participate (87.1% response rate). Among these, 698 parents returned the survey with the *EFB-K* completed. After data screening, 12 respondents were excluded for not being parents, eight for not reporting their relationship to the child, and one because the child did not live with the participating parent. The characteristics of the remaining 677 parents are presented in Table 1. Among these parents, 654 children also completed the survey.

**Table 1:** Demographic and clinical characteristics of the participants.

	*N*	Mean (±SD) or *n* (%)^a^
Parents	677	
Gender, female	677	522 (77.1%)
Age, years	672	43.9 (±6.3)
Education, ≥ 12 years	677	354 (52.3%)
Birthplace, Germany	676	578 (85.5%)
Employment status, full‐time/part‐time	672	263 (39.1%)/319 (47.5%)
Relationship status, together, single, new partner	675	519 (76.9%)/91 (13.5%)/65 (9.6%)
PHQ‐9, total	673	6.28 (±4.62)
PAID‐parent, mean		3.14 (±1.01)
Child characteristics		
Age, years	677	12.4 (±2.9)
Gender, female	642	287 (44.7%)
Diabetes duration, years	676	5.4 (±3.8)
Current HbA1c, %/mmol/mol	618	7.5 (±0.97)/58.6 (±10.6)
Time in range (70–180 mg/dL; 3.9–10.0 mmol/L) last 14 days, %	457	60.2 (±17.6)
Insulin pump use, yes	675	401 (59.4%)
CGM use, yes	675	562 (83.3%)
Children	654	
Number of glucose checks per day	653	
0		4 (0.6%)
1–2		28 (4.3%)
3–4		91 (13.9%)
5–6		149(22.8%)
7–8		141 (21.6%)
≥ 9		240 (36.8%)
PAID‐C/PAID‐T, mean	649	2.35 (±0.97)
HRQoL, *T*‐score	640	51.61 (±10.15)

^a^Percentages were calculated excluding missing values.

Parents had a mean age of 43.9 (± 6.3) years, were predominantly mothers (77.1%), and mostly born in Germany (85.5%). Children were on average 12.4 (± 2.8) years old, with a mean diabetes duration of 5.4 (± 3.8) years. Approximately 29% of the children met the recommended HbA1c level of < 7% (53 mmol/mol) and 26.9% met the TiR recommendation of > 70% [2, 3].

### 3.1. Parenting Behavior and Demographic Characteristics

The mean *overreactivity* score was 3.22 (± 1.04) and the mean *laxness* score was 2.93 (± 0.92), with the overall mean *EFB-K* score of 3.03 (± 0.77). Using the suggested cutoffs (≥ 4.34 for *overreactivity* and ≥ 3.43 for *laxness*), 88 (13.0%) parents met criteria for *overreactivity* and 182 (26.9%) for *laxness*.

Parenting practices did not significantly differ between mothers and fathers (*overreactivity*: mothers = 3.26 [±1.07] vs. fathers = 3.09 [±0.90], *t*(675) = 1.85, *p* = 0.065; *laxness*: mothers = 2.90 [±0.93] vs. fathers = 3.01 [±0.87], *t*(675) = −1.34, *p* = 0.180). Parents with ≥ 12 years of education reported significantly lower *laxness* (2.83 [± 0.86] than those with < 12 years (3.03 [± 0.97]), *t*(675) = 2.85, *p* = 0.005, Cohen^′^s *d* = 0.22). German‐born parents had lower *laxness* scores (2.88 [± 0.91]) compared with non‐German–born parents (3.15 [± 0.97], *t*(674) = −2.69, *p* = 0.007, *d* = 0.29). Mean *overreactivity* scores differed by parents′ relationship status (*F*(2, 672) = 4.16, *p* = 0.016). Parents with a new partner reported low *overreactivity* than those living with the child′s other parent (mean difference = −0.38, 95% CI [−0.71, −0.06]. *p* = 0.015) and single parents (mean difference = −0.41, 95% CI [0.81, −0.01], *p* = 0.042). Parent age was not significantly correlated with either parenting behavior.

**Figure 1:**
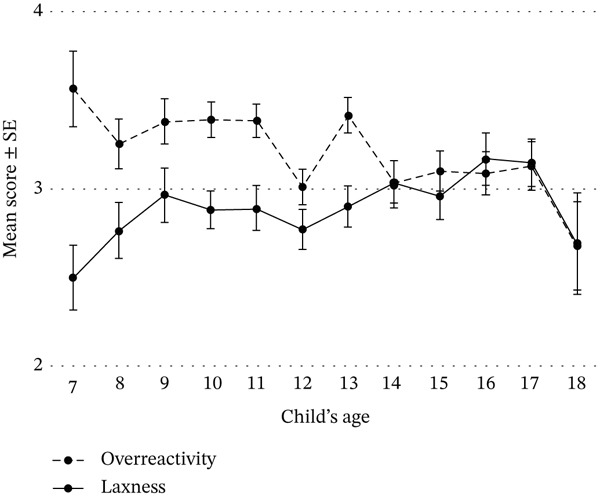
Parents′ mean *overreactivity* and *laxness* scores by child age.

Parenting behavior did not differ by child gender: *overreactivity*: girls = 3.16 (±1.04) versus boys = 3.25 (±1.02), *t*(640) = 1.14, *p* = 0.254; *laxness*: girls = 2.89 (±0.92)  versus 2.92 (± 0.91), *t*(640) = 0.43, *p* = 0.668. Child age, however, showed small but significant correlations with both *overreactivity* (*r* = −0.114, *p* = 0.003) and *laxness* (*r* = 0.104, *p* = 0.007) such that, as the child′s age increased, parents reported lower *overreactivity* and higher *laxness* (see Figure 1). Parenting behavior was not significantly associated with insulin pump use (overreactivity: no pump = 3.27 [1.01] vs. pump 3.18 [1.04]; laxness: no pump = 2.99 [0.89] vs. pump = 2.87 [0.94]) or CGM use (overreactivity: no CGM = 3.16 [1.00] vs. CGM = 3.23 [1.04]; laxness: no CGM = 3.03 [0.94] vs. CGM = 2.91 [0.92]). Diabetes duration was not correlated with either behavior (overreactivity: *r* = −0.058, *p* = 0.130; laxness: *r* = 0.033, *p* = 0.393).

### 3.2. Correlation Between Parenting Behavior and Psychosocial Variables

Children′s *HRQoL* was inversely correlated with parental *overreactivity* (*r* = −0.135, *p* < 0.001) (Table 2). Similarly, parents reporting higher *overreactivity* and *laxness* also reported greater diabetes‐related distress of their own (*r* = 0.335, *p* < 0.001; *r* = 0.119, *p* < 0.01, respectively) and more depressive symptoms (*r* = 0.314, *p* < 0.001; *r* = 0.100, *p* < 0.01, respectively).

**Table 2:** Correlation of parenting behaviors with clinical and psychological measures.

		Overreactivity			Laxness		
Variables	*N*	*r*	95% CI		*r*	95% CI	
Glucose checks per day	652	−0.025	−0.110	0.040	−0.134	−0.206	−0.057
HbA1c	618	−0.021	−0.099	0.058	0.034	−0.045	0.113
Time in range	457	−0.025	−0.117	0.066	−0.053	−0.144	0.039
Child HRQoL^a^	640	−0.135	−0.211	−0.058	−0.012	−0.090	0.065
Child diabetes distress^b^	649	0.197	0.122	0.270	0.122	0.045	0.197
Parent diabetes distress^c^	672	0.335	0.266	0.400	0.119	0.044	0.193
Parent depressive symptoms^d^	673	0.314	0.245	0.381	0.100	0.025	0.174

^a^The KIDSCREEN‐10 index (self‐report).

^b^The Problem Areas in Diabetes (PAID) Child/Teen versions.

^c^The Problem Areas in Diabetes (PAID) Parent versions.

^d^The Patient Health Questionnaire (PHQ)‐9.

### 3.3. Predicting Child′s Glucose Monitoring Frequency, HbA1c, and Diabetes Distress Using Parenting Behaviors

In the hierarchical regression predicting children′s glucose monitoring frequency, higher parental *laxness* was associated with fewer daily glucose checks (Table 3). Additional significant predictors were child age, insulin pump use, CGM use, parental education level, and child *HRQoL*. In the model predicting HbA1c, parenting behaviors were not significant predictors. Significant factors included diabetes duration, CGM use, parental education, parental relationship status, and child *HRQoL*. In the model predicting children′s diabetes distress, both *overreactivity* and *laxness* were significantly associated with higher diabetes distress. Other significant factors included parental education and child *HRQoL*.

**Table 3:** Hierarchical regression analyses predicting glucose checking frequency, HbA1c, and diabetes distress in children and adolescents with T1D.

Model	Predictor	*R* ^2^	*R* ^2^ *Δ*	*B*	*S* *E* *B*	*β*	*p*
Glucose checks, *n* = 631							
	Child age	0.109 ^∗∗∗^		−0.047	0.020	−0.107	0.019
	Child gender, female			−0.016	0.097	−0.006	0.868
	Duration			0.002	0.016	0.006	0.903
	Insulin pump, yes			−0.316	0.109	−0.124	0.004
	CGM, yes			0.564	0.134	0.164	< 0.001
	Parent education, ≥ 12 years			0.303	0.096	0.121	0.002
	HRQoL‐Child			0.020	0.005	0.163	< 0.001
	*Overreactivity*	0.120 ^∗∗∗^	0.011 ^∗^	0.001	0.049	0.000	0.992
	*Laxness*			−0.149	0.056	−0.107	0.008

HbA1c, *n* = 580							
	Child age	0.152 ^∗∗∗^		0.026	0.016	0.077	0.098
	Child gender, female			0.083	0.076	0.043	0.275
	Duration			0.044	0.012	0.176	< 0.001
	Insulin pump, yes			0.035	0.086	0.018	0.683
	CGM, yes			−0.346	0.106	−0.131	0.001
	Parent education, ≥ 12 years			−0.152	0.076	−0.079	0.045
	New partner parent			0.270	0.126	0.084	0.033
	Single parent			0.269	0.112	0.095	0.017
	HRQoL‐Child			−0.016	0.004	−0.174	< 0.001
	*Overreactivity*	0.153 ^∗∗∗^	0.001	−0.025	0.038	−0.028	0.510
	*Laxness*			0.016	0.043	0.015	0.713

Diabetes distress, *n* = 627							
	Child age			0.009	0.013	0.027	0.475
	Child gender, female			0.083	0.063	0.043	0.189
	Duration	0.371 ^∗∗∗^		−0.003	0.010	−0.010	0.807
	Insulin pump, yes			0.046	0.071	0.023	0.516
	CGM, yes			−0.081	0.088	−0.030	0.357
	Parent education, ≥12 years			−0.137	0.063	−0.070	0.030
	New partner parent			0.130	0.106	0.040	0.218
	Single parent			0.031	0.092	0.011	0.735
	HRQoL‐Child			−0.055	0.003	−0.575	< 0.001
	*Overreactivity*	0.387 ^∗∗∗^	0.016 ^∗∗∗^	0.090	0.032	0.096	0.005
	*Laxness*			0.071	0.036	0.066	0.050

^∗^ *p* < 0.05.  ^∗∗∗^ *p* < 0.001.

Age‐stratified analyses revealed that among younger children (7–10 years), parental *laxness* was associated with reduced glucose monitoring and *overreactivity* predicted higher diabetes distress (Table 4). Among early adolescents (11–14 years), no significant associations emerged for three analyses. Among older adolescents (15–18 years), *laxness* was linked to fewer glucose checks, higher HbA1c, and increased diabetes distress.

**Table 4:** Backward regression analyses by age group examining contributing factors to glucose checking frequency, HbA1c, and diabetes distress in children and adolescents with T1D.

Model	Independent variable	Age group	*B*	SE *B*	*β*	*p*	95% CI	
Glucose checks								
	Constant	7–10	4.215	0.563		< 0.001	3.104	5.325
	Child HRQoL	*n* = 183	0.028	0.009	0.221	0.002	0.010	0.046
	*Laxness*		−0.285	0.106	−0.192	0.008	−0.494	−0.076
	Constant	11–14	3.825	0.369		< 0.001	3.099	4.551
	Insulin pump, yes	*n* = 269	−0.312	0.140	−0.133	0.026	−0.587	−0.037
	HRQoL‐Child		0.022	0.007	0.188	0.002	0.008	0.035
	Constant	15–18	0.389	1.480		0.793	−2.533	3.311
	Child age	*n* = 179	0.248	0.089	0.186	0.006	0.072	0.424
	CGM, yes		0.949	0.221	0.291	< 0.001	0.513	1.386
	Parent education, ≥ 12 years		0.374	0.172	0.149	0.032	0.033	0.714
	*Laxness*		−0.294	0.090	−0.224	0.001	−0.472	−0.117
HbA1c								
	Constant	7–10	5.643	0.564		< 0.001	4.529	6.758
	Child age	*n* = 161	0.173	0.062	0.217	0.006	0.051	0.296
	Constant	11–14	8.089	0.327		< 0.001	7.445	8.732
	Duration	*n* = 245	0.078	0.017	0.276	< 0.001	0.046	0.111
	CGM, yes		−0.348	0.150	−0.138	0.022	−0.644	−0.051
	Single parent		0.538	0.178	0.181	0.003	0.187	0.889
	HRQoL‐Child		−0.014	0.006	−0.144	0.016	−0.025	−0.003
	Constant	15–18	13.280	1.364		< 0.001	10.588	15.973
	Child age	*n* = 174	−0.289	0.078	−0.265	< 0.001	−0.443	−0.134
	Duration		0.038	0.017	0.156	0.031	0.004	0.073
	CGM, yes		−0.415	0.190	−0.151	0.031	−0.791	−0.039
	HRQoL‐Child		−0.028	0.007	−0.268	< 0.001	−0.042	−0.014
	*Laxness*		0.167	0.077	0.153	0.031	0.016	0.318
Diabetes distress								
	Constant	7–10	4.156	0.415		< 0.001	3.336	4.976
	Child gender, female	*n* = 179	0.233	0.114	0.123	0.042	0.008	0.458
	HRQoL‐Child		−0.050	0.005	−0.566	< 0.001	−0.061	−0.040
	*Overreactivity*		0.131	0.057	0.138	0.023	0.018	0.244
	Constant	11–14	5.455	0.233		< 0.001	4.995	5.915
	Child HRQoL	*n* = 268	−0.060	0.004	−0.638	< 0.001	−0.069	−0.051
	Constant	15–18	4.518	0.392		< 0.001	3.745	5.291
	HRQoL‐Child	*n* = 180	−0.055	0.006	−0.529	< 0.001	−0.068	−0.043
	*Laxness*		0.244	0.064	0.230	< 0.001	0.117	0.371

*Note:* *R* ^2^ values for glucose checks models by *a* *g* *e* *g* *r* *o* *u* *p* (7–10, 11–14, 15–18) = 0.083, 0.054, and 0.228, respectively; the *H* *b* *A*1*c* *m* *o* *d* *e* *l* *s* = 0.047, 0.179, and 0.197; and the *d* *i* *a* *b* *e* *t* *e* *s* *d* *i* *s* *t* *r* *e* *s* *s* *m* *o* *d* *e* *l* *s* = 0.377, 0.407, and 0.352.

## 4. Discussion

This study is the first to examine ineffective parenting behaviors in discipline situations, specifically *overreactivity* and *laxness*, in a large sample of parents of children and adolescents with T1D in Germany. We explored their associations with demographic, clinical, and psychosocial outcomes. Consistent with expectations, both *overreactivity* and *laxness* were associated with diabetes‐specific distress in children and parents. Greater parental *laxness* was associated with reduced glucose monitoring behavior. Moreover, both dysfunctional parental practices were positively correlated with the parent′s own depressive symptoms. Together, these results highlight the importance of parenting behavior for the well‐being of the parent–child dyad and for children′s diabetes self‐management.

### 4.1. Parenting Behavior and Demographic Characteristics

Most parents reported adaptive discipline strategies, while approximately one‐quarter met the cutoff for high *laxness* and 13% for *overreactivity*, indicating a small subgroup showing problematic disciplinary patterns. The predominance of mothers among participants reflects common caregiving patterns in diabetes management [36, 37]. Nevertheless, no significant gender differences emerged in parenting behaviors suggesting that, when fathers are actively involved in diabetes care, they also use similar discipline strategies as mothers. Parental behavior did not differ by the child′s gender, contrary to previous findings [11]. Differences in measurement approaches may explain this discrepancy. Tilden et al. used an observational assessment to examine the effects of intrusive parenting in diabetes‐related conflict situations [11]. The *EFB-K* questionnaire assesses self‐reported general disciplinary behavior over the last 2 months.

Interestingly, parents in a new relationship reported lower *overreactivity* toward their child than those living with the other parent or single parents. This possibly reflects reduced relationship stress for parents in a new relationship. Prior research has shown that parental stress is closely linked to parenting behavior [38, 39]. When experiencing high levels of stress, parents seem to be more likely to use dysfunctional parenting behavior. Establishing parenting routines for disciplinary situations can help to implement positive parenting behavior and simultaneously reduce parenting stress [40].

Parenting behaviors were related to children′s age. Parents of older children reported less *overreactivity* and more *laxness*, consistent with increasing autonomy and declining parental control during adolescence. While permissive parenting can support positive psychological development in older children [19, 41], *laxness* characterized by inconsistent rule setting and enforcement in disciplinary situations has been associated with increased emotional and behavioral problems [19, 42, 43]. Therefore, maintaining a balance between supporting autonomy and enforcing consistent parenting appears essential for promoting healthy development and effective diabetes management in adolescents.

There were no differences in parenting behavior based on the child′s therapy type in this study, suggesting that parental overreactivity and laxness may be more closely linked to the demands of diabetes management rather than to a specific treatment modality. Children in this sample were treated with either insulin pump or pen therapy, both of which require consistent monitoring and supervision, particularly for younger children, and these demands often continue into adolescence. In contrast, automated insulin delivery (AID) systems may alter parent–child interactions and parenting behaviors around diabetes care due to reduced management demand. Future studies should examine parenting behaviors in families using AID systems compared with other treatment modalities, particularly in diabetes care contexts, to develop targeted parenting interventions and clinical recommendations.

### 4.2. Parenting and Diabetes Outcomes

Contrary to our hypothesis, neither *overreactivity* nor *laxness* was directly correlated with HbA1c and TiR. Our overall regression model of HbA1c was not significantly affected by parenting behaviors. Previous studies have reported mixed findings regarding the relationship between parenting behavior and HbA1c [9, 11, 44]. One possible explanation for this absence of association may reflect the mitigating effect of intense diabetes education offered in respective pediatric diabetes centers and the use of modern diabetes technologies such as CGM and insulin pumps, which can enhance treatment adherence independent of parenting practices [11, 44]. As the use of AID systems has increased in recent years, the influence of parenting on glycemic control may be diminishing although it continues to affect the child′s adherence behaviors and mental well‐being.

Consistent with our findings, higher parental *laxness* was associated with less frequent glucose monitoring behaviors in children and adolescents. This suggests that inconsistent discipline may still undermine daily self‐management tasks even when automated systems facilitate other aspects of diabetes care. For instance, *laxness* in parents may weaken routines, accountability, self‐discipline, and self‐motivation in children. The effects on self‐management behavior examined in this study support the importance of implementing boundaries, consistent supervision and consequences in parenting.

Both *overreactivity* and *laxness* were associated with psychosocial distress in parents and children. Parents reporting more dysfunctional parenting behaviors also reported higher diabetes‐related distress and more depressive symptoms. This aligns with other studies linking parental depression and stress to less effective parenting [23, 43, 45]. Children′s diabetes distress and *HRQoL* were also closely linked to parental discipline. There is strong evidence in the literature that parenting behavior is a predictor for children′s and adolescents′ emotional adjustment, such as internalizing and externalizing problem behavior and overall mental health [11, 19]. The results of this study confirmed the impact of distinct parenting behavior not only on children′s well‐being but also on parental mental health.

### 4.3. Developmental Differences and Parenting

Among younger children (7–10 years), parental *laxness* was associated with reduced frequency of glucose checks, and *overreactivity* predicted of higher diabetes‐specific distress, indicating both inconsistent and harsh disciplinary behaviors have negative influence on children′s early diabetes management and emotional well‐being [19]. Among early adolescents (11–14 years), no significant association emerged, possibly reflecting a complicated transitional phase. At the time of early puberty, child′s physical and emotional developments can differ profoundly [46, 47], which may lead to varied parenting patterns. Moreover, diabetes management responsibilities increasingly shift from parent to child as German children enter secondary school. In older adolescents (15–18 years), parental *laxness*, which is more common parenting pattern in this age group, was linked to fewer glucose checks, higher HbA1c, and increased diabetes distress. Our finding mirrors previous research showing an association between a permissive parenting style and poor adherence in adolescents [8]. Together, this demonstrates the importance of continuous parental involvement by consistent and effective limit‐setting and enforcement of set rules even for older adolescents.

### 4.4. Clinical Implications

The results of this study highlight the impact of distinct parenting behavior on both child and parent well‐being. Promoting consistent, responsive, and autonomy‐supportive parenting may enhance diabetes self‐management and reduce family distress. Parent training programs grounded in cognitive‐behavioral or mindfulness‐based framework incorporating healthy parenting behavior in disciplinary situations may improve emotional regulation, reduce overreaction, and strengthen consistency. Existing parent‐focused interventions teach helpful strategies to support independence in children and adolescents with T1D while implementing positive strategies for consistent behavior in disciplinary situations have shown efficacy [48–50]. These interventions have the potential to reduce negative effects on children and adolescents while increasing parental well‐being [48–50]. Regular screening for parental diabetes distress and depressive symptoms during routine visits may further help identify families in need of support.

### 4.5. Strengths and Limitations

Key strengths of this study include its large multicenter design, use of validated psychological instruments, and age‐stratified analyses. Limitations include its cross‐sectional design, which limits establishing causality. Findings in this study were based on self‐report of parents, and this may have introduced response bias such as an underestimation of dysfunctional parenting behavior due to social desirability. To address these limitations, future studies should employ longitudinal designs and include observational measures to clarify mechanisms and reduce reporting bias. The predominance of mothers may have reduced the generalizability of the findings to fathers. Furthermore, the lack of normative *EFB-K* data and evaluation studies for older children limits the interpretation of cutoffs used in this study. Finally, although age groups were stratified based on developmental and environmental considerations, the middle age group (11–14 years) is characterized by substantial heterogeneity in pubertal and developmental stages. Variability in adolescents′ hormonal and physical development, maturity, and self‐management abilities may have differentially influenced parenting behavior and may partly explain the lack of clear pattern within this age group.

### 4.6. Conclusions

Dysfunctional parenting behaviors are associated with poorer psychosocial outcomes and reduced diabetes self‐management in children and adolescents. The results of this study suggest that parental support needs to be tailored for children′s developmental stage in pediatric diabetes care. Overreaction in parents of younger children and inconsistency or ineffective limit‐setting for adolescents may contribute to increased distress and reduced family functioning. Interventions that foster emotional regulation, consistent discipline, autonomy support, and healthy communication may enhance both family well‐being and diabetes outcomes, especially for highly burdened parents.

## Author Contributions

Alessa Thomas and Su‐Jong Kim‐Dorner shared first authorship.

## Funding

This study was supported by the Deutsche Diabetes Gesellschaft (10.13039/501100010215). Open Access funding enabled and organized by Projekt DEAL.

## Conflicts of Interest

The authors declare no conflicts of interest.

## Data Availability

The data that support the findings of this study are available from the corresponding author upon reasonable request.
